# Do family physicians advise younger people on cardiovascular disease prevention? A cross-sectional study from Slovenia

**DOI:** 10.1186/1471-2296-14-82

**Published:** 2013-06-14

**Authors:** Davorina Petek, Rok Platinovsek, Zalika Klemenc-Ketis, Janko Kersnik

**Affiliations:** 1 Department of Family Medicine, Medical Faculty, University of Ljubljana, Poljanski nasip 58, 1000, Ljubljana, Slovenia; 2 Faculty of Social Sciences, University of Ljubljana, Kardeljeva ploscad 5, 1000, Ljubljana, Slovenia; 3 Department of Family Medicine, Medical Faculty, University of Maribor, Slomskov trg 15, 2000, Maribor, Slovenia

**Keywords:** Primary prevention, Cardiovascular diseases, Family practice, Counselling, Multilevel analysis

## Abstract

**Background:**

One of the main family practice interventions in the younger healthy population is advice on how to keep or develop a healthy lifestyle. In this study we explored the level of counselling regarding healthy lifestyle by family physicians and the factors associated with it.

**Methods:**

A cross-sectional study with a random sample of 36 family practices, stratified by size and location. Each practice included up to 40 people aged 18–45 with low/medium risk for cardiovascular disease (CVD). Data were obtained by patient and practice questionnaires and semi-structured interviews. Several predictors on the patient and practice level for received advice in seven areas of CVD prevention were applied in corresponding models using a two-level logistic regression analysis.

**Results:**

Less than half of the eligible people received advice for the presented risk factors and the majority of them found it useful. Practices with medium patient list-sizes showed consistently higher level of advice in all areas of CVD prevention. Independent predictors for receiving advice on cholesterol management were patients’ higher weight (regression coefficient 0.04, p=0.03), urban location of practice (regression coefficient 0.92, p=0.04), organisation of education by the practice (regression coefficient 0.47, p=0.01) and practice list size (regression coefficient 6.04, p=0.04). Patients who self-assessed their health poorly more frequently received advice on smoking (regression coefficient −0.26, p=0.03). Hypertensive patients received written information more often (regression coefficient 0.66, p=0.04). People with increased weight more often received advice for children’s lifestyle (regression coefficient 0.06, p=0.03). We did not find associations with patient or practice characteristics and advice regarding weight and physical activity. We did not find a common pattern of predictors for advice.

**Conclusions:**

Counselling for risk diseases such as increased cholesterol is more frequently provided than basic lifestyle counselling. We found some doctors and practice factors associated with counselling behaviour, but the majority has to be explained by further studies.

## Background

Prevention of cardiovascular diseases for high risk population is a traditional task of family physicians as part of the comprehensive approach and community orientation [1]. There is ample evidence that multiple interventions lower mortality in high risk groups but the evidence for health-promoting activities in the general population is still not unanimous [2] and is the subject of extensive research activity [3].

In primary cardiovascular diseases (CVD) prevention, a substantial part of the older generation is considered to have high cardiovascular risk even with only a moderate elevation of modifiable risk factors. As a consequence, interventions are focused on this generation and not on the younger population, for whom cardiovascular risk is also assessed less often. Primary prevention of the younger population has traditionally been focused on the prevention of transmissible diseases and was typically provided through public health agencies. In recent years, cardiovascular prevention also became the task of family physicians who can provide individual counselling and use other strategies for lifestyle modification. European guidelines on cardiovascular prevention emphasise objectives of cardiovascular prevention in the younger population: to retain a healthy lifestyle and to improve risky behaviour where necessary [4].

According to European guidelines [4], primary prevention in the broad sense of counselling to retain a healthy lifestyle and improve risky behaviour should also be directed at healthy people without any known risk factors. This is a feature of the holistic and comprehensive approach typical for family medicine. Furthermore, advice on healthy lifestyle cannot be given without assessing one’s lifestyle first. Therefore, GPs should advise all patients on a healthy lifestyle - regardless of their health status and the presence of risk factors.

For the younger population some national guidelines even suggest to intervene at a lower risk threshold [5]. Despite recommendations, many physicians feel that healthcare’s resources and budget are insufficient for all primary prevention activities and cannot meet the needs of this group of people [6].

Many studies on cardiovascular prevention have focused on the population older than 40 or 50 years [7,8] or on a wide age range population [9] and have dealt with their risk assessment. Only a few studies have addressed cardiovascular prevention for young people [10]. Attitudes of people towards lifestyle change, the role of their family doctor [11], patient expectations and received counselling [12] have been assessed in the whole population, not taking into account any age differences.

Younger groups of people usually come for a consultation when they need a health service for acute health problem and are classified as less frequent visitors. Young people are usually not included in routine preventive programs. Slovenia is one of the few European countries that launched a National Programme of Cardiovascular Prevention already in 2001. One of its main critiques was that eligible people groups were too old (men 35–65, women 45–70 years old) in terms of long-term primary prevention impact.

Attitudes and practice toward preventive activities differ also between the family physicians themselves; they depend on their own lifestyle [13] and on their practice characteristics such as a heavy workload.

In our study, we aimed to explore the level of counselling that young people receive in family medicine practices and their experience with the received counselling, and to determine factors that can influence healthy lifestyle counselling provision.

## Methods

The study was a part of the international European Practice Assessment of Cardiovascular risk management (Epa-Cardio) study, which involved nine European countries. Here, we present results of the Slovenian sample. The detailed description of methodology has already been published elsewhere [14].

### Subjects

A random sample of 36 family medicine practices, stratified by size (small: up to two full-time equivalent (FTE) working family physicians on the same location, large: more than two FTE family physicians) and location (urban: more than 30,000 habitants, rural: 30,000 habitants or less) were included in the study. Out of 56 invited practices, 36 agreed to participate (response rate of 64.3%).

In each practice, we aimed to include a random sample of 40 people (age 18 to 45) from their list of registered patients. Eligible people were those without any chronic cardiovascular diseases. Patients with a diagnosed arterial hypertension or hypercholesterolemia but not assessed by the family physician as being at high risk for cardiovascular diseases were eligible for inclusion as well. Cardiovascular risk assessment was defined by using the Framingham score system, which is by national agreement a compulsory tool for CVD risk assessment in the country. It provides scores from 0 to over 40 on the basis of patients’ age, sex, smoking history, systolic blood pressure and cholesterol levels. The scores are calculated automatically by a computer programme as part of each patient’s record keeping system after entering these data. Patients with scores from 0 to 20 on a Framingham scale were eligible for inclusion. It is mostly useful in age groups over 40. People were invited to take part in the study by phone and mail. An introductory letter, the letter of agreement to participate in the study, signed by their physician and practicing nurse were sent out.

Out of 1,440 invited people, 953 of them returned the questionnaires. Later on we excluded 16 people because of missing data and performed a final analysis on 937 questionnaires by list-wise exclusion (response rate of 65.0%).

### Questionnaire

Each enrolled participant filled in the questionnaire that consisted of basic demographic data (gender, age, education, marital and employment status), self-assessment of health (using a five-point Likert-type scale ranging from excellent (5 points) to poor (1 point), length of attachment to the practice and frequency of yearly attendance of the practice. In the second part of the questionnaire, the participants stated whether they received advice on risk factors for CVD and lifestyle and gave their opinion on the usefulness of this advice. Some questions on whether CVD advice was received were not administered to all respondents but rather to a smaller subgroup. The question on smoking advice was only administered to smokers, ex-smokers or occasional smokers and the question on children’s lifestyle was only administered to respondents with children. All patients also filled out the questionnaire on their lifestyle. The following validated questionnaires were used for this purpose: physical activity (RAPA questionnaire) [15], eating habits [16,17] and smoking status (MID-SIZED Model questionnaire) [18].

Family physicians from each included practice filled out the questionnaire on practice characteristics. Additionally, the main researcher (DP) performed a semi-structured interview with all of the physicians. The questionnaire included questions on practice-led contacts for prevention (system for recalling people for CVD prevention), clinical information system (computer-supported patient file system), case finding methods to detect people with cardiovascular risk factors, existing procedures for smoking cessation, work in community (participation of physicians and nurses in public healthcare programmes on lifestyle), education of GPs and nurses on CVD in the last year and registered patients’ list-size.

The study was approved by the Slovenian National Committee on Medical Ethics (No. 87/11/07).

### Statistical methods

The Epa-Cardio data cannot be analyzed with classical methods because these methods assume that the units were sampled independently. This assumption is violated in our case because people were sampled in clusters (practices). People attending the same practice are likely to be more similar than people attending different practices. This clustering requires the use of multilevel analysis [19,20], which allows for correct estimation of the standard errors of predictors in the explanatory model. This is especially important since we have a number of predictors that are measured at the practice level whose standard errors would be grossly underestimated with classical methods of analysis. The statistical analysis is focused on whether the participant received advice in one of the seven cardiovascular diseases prevention areas. Because the response was dichotomous (advice received/not received), we applied a two-level logistic regression to the data. We fit the so-called random intercept model seven times – to each of the areas of advice that the people could receive. The response variables and patient-level predictors are described in the following section. A specific part of our sample - males between 35 and 45 years are included in the Slovenian National Preventive Programme, which is why we hypothesized that this group would receive advice on lifestyle more often than the rest of the population. A corresponding dummy-variable was included in the analyses.

The software used to perform the estimation was the package lme4 [21] for R (R Development Core Team 2011) [22].

## Results

### Description of the sample

The majority (23 or 63.9%) of the participating practices were small practices (employed no more than two GPs) and located in rural areas (26 practices or 72.2%). The mean age of people was 35.2 (SD 8,1) years. The basic demographic structure of the participating people is given in Table 1.

**Table 1:** Demographic, behavioural and health self-assessment characteristics of 937 patients aged from 18-45 years with low/medium cardiovascular risk

**Demographic characteristics**	**No. (%) of patients**
**Gender**	
Men	384 (41.0)
Women	528 (56.4)
Missing	25 (2.7)
**Age**	
18-30	266 (28.4)
31-40	388 (41.4)
41-45	261 (27.9)
Missing	22 (2.3)
**Education**	
Primary school or less	134 (14.3)
Secondary school	453 (48.3)
University	313 (33.4)
Missing	37 (3.9)
**Employment status**	
Unemployed	116 (12.4)
Employed	788 (84.1)
Missing	33 (3.5)
**Marital status**	
Married, cohabiting	649 (69.3)
Single, divorced, widowed	264 (28.2)
Missing	24 (2.6)
**Self assessment of health**	
Poor	37 (3.9)
Fair	139 (14.8)
Good	386 (41.2)
Very good	276 (29.5)
Excellent	71 (7.6)
Missing	28 (3.0)
**Practice attendance (years)**	
≤ 2	112 (12.0)
3-7	226 (24.1)
8-12	169 (18.0)
>13	401 (42.8)
Missing	29 (3.1)
**Visit frequency/year (n=918)**	
0-1	212 (22.6)
2-3	384 (41.0)
4-5	177 (18.9)
6-7	68 (7.3)
≥ 8	77 (8.2)
Missing	19 (2.0)

### Lifestyle/ risk factors of the respondents

According to the RAPA questionnaire, less than half of the sample of 850 people had adequate aerobic physical activity (384 respondents, 45.2%) and an even lower percent of the people were also performing exercises for stretching and for muscular strength (240 respondents, 28.5% of the sample).

The average score on the REAP-S questionnaire for healthy diets of the respondents was 26.5 (SD=4.0). The lowest possible score on this item was 12 points and the highest score 39 with higher scores indicating a healthier diet.

312 respondents (33.3%) were overweight (BMI 25–30 kg/m2) and 138 (14.7%) were obese (BMI >30 kg/m2). 89 (9.5%) patients claimed that they had hypertension and 87 (9.3%) patients stated that they had hypercholesterolemia.

424 (45.2%) of respondents stated that they never smoked cigarettes; others were current, occassional or past smokers.

### Advice on lifestyle

**Figure 1:**
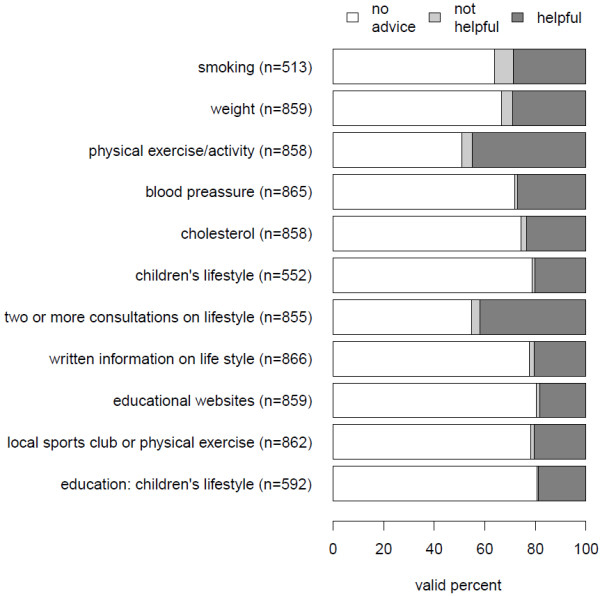
**Advice and avowed helpfulness of advice- valid row percentages in the sample of younger people in national part of the Epa-Cardio study.** Legend: Number of given advice in different areas differs due to missing values in the questionnaire and due to analysis of two subgroups of patients (smokers/ex-smokers, people with children).

Figure 1 shows how many respondents recall being given advice in seven CVD areas. Less than half of the eligible respondents received advice for the presented risk factors. In the two cases where the subgroups of (ex)smokers/occasional smokers and people with children were analysed, the percentages in Figure 1 pertain to the narrower subgroup as is also reflected in the lower number of cases.

The questionnaire also inquired whether the respondents found the advice to be helpful or not. Because the great majority of respondents reported the advice to be helpful, we performed the analysis by merging the categories “not helpful” and “helpful” and performed the multilevel analyses on the resulting dichotomous (rather that multinomial) variable discriminating only between whether advice was given or not.

**Figure 2:**
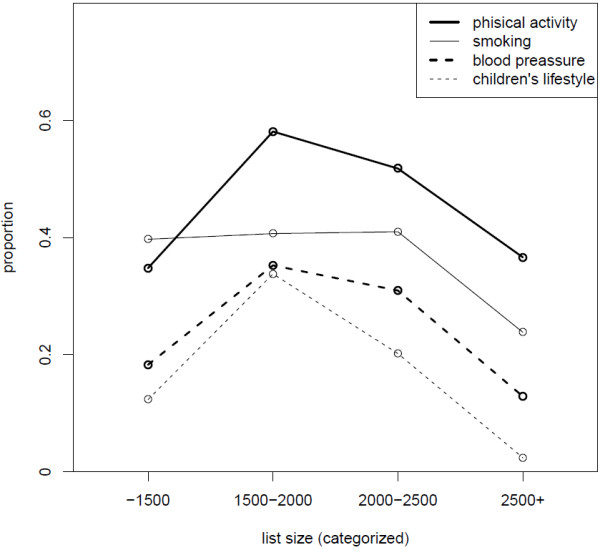
Proportion of advice on smoking, blood pressure, physical activity and children’s lifestyle according to patient list size.

**Figure 3:**
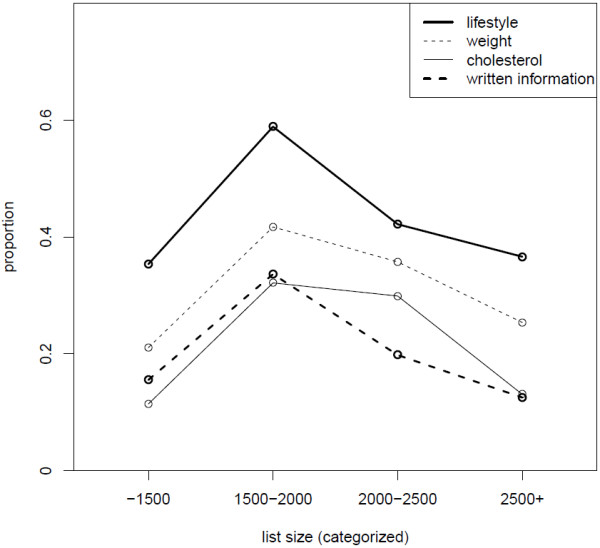
Proportion of complex lifestyle advice, advice on weight, cholesterol and given written information according to patient list size.

While conducting preliminary bivariate analyses, we encountered a curvilinear association between the patient list size (the total number of patients on the practice list of one GP) and the proportion of respondents who reported receiving CVD advice. Figures 2 and 3 depict the proportion of respondents who received advice as a function of the list size categorized into four categories. The proportion of respondents who received advice is lowest in the high and low numbers of people on the list, while this proportion is higher in the middle two categories of the list size. This trend is remarkably consistent across all eight areas of CVD advice.

### Factors related to received advice for prevention of CVD

Table 2 shows association of several predictor variables on patient and practice level with received preventive advice.

**Table 2:** Multilevel logistic regression analysis of patient and practice factors on received advice for prevention of CVD in the sample of younger people (regression coefficient above; statistical significance below in each cell)

	**Smoking**	**Weight**	**Physical activity**	**Cholesterol**	**Lifestyle**	**Children lifestyle**	**Written info**
	**N= 478**	**N=775**	**N=779**	**N=770**	**N=777**	**N=501**	**N=774**
**intercept**	**−3.89**	**-3.52**	**-3.45**	**-9.69**	**-3.12**	**-20.56**	**-3.32**
	**0.16**	**0.17**	**0.12**	**0.00**	**0.22**	**0.00**	**0.29**
**Gender (female)**	0.36	-0.07	0.07	-0.01	-0.20	0.14	0.23
	0.11	0.68	0.67	0.98	0.26	0.60	0.27
**age**	-0.01	0.01	0.00	-0.01	0.01	**-0.06**	-0.02
	0.75	0.29	0.89	0.70	0.21	**0.01**	0.19
**Male 35-45 years**	1.42	1.17	0.81	-1.08	-0.04	1.04	-0.36
	0.05	0.05	0.14	0.23	0.95	0.66	0.05
**education**	-0.03	0.08	0.04	0.27	-0.15	-0.06	-0.20
	0.87	0.54	0.77	0.09	0.25	0.74	0.09
**Employment (not employed)**	0.14	0.05	-0.07	0.46	0.34	-0.10	0.36
	0.65	0.85	0.76	0.10	0.17	0.81	0.10
**Marital status (not married)**^**1**^	'0.06	'0.01	0.00	0.09	-0.11	-0.56	-0.15
	0.82	0.97	0.99	0.71	0.58	0.07	0.51
**Self assessment of health**^**2**^	**-0.26**	-0.08	0.00	-0.06	0.14	0.11	0.08
	**0.03**	0.36	0.96	0.60	0.12	0.47	0.47
**BMI**^**3**^	0.03	0.01	0.00	**0.04**	-0.01	**0.06**	0.03
	0.14	0.53	0.99	**0.03**	0.47	**0.03**	0.15
**Hypertension**^**4**^	0.16	0.20	-0.19	-0.08	0.32	0.37	**0.66**
	0.67	0.50	0.50	0.82	0.27	0.38	**0.04**
**Practice size (large)**	-0.03	-0.04	0.01	0.06	0.33	0.04	0.00
	0.93	0.89	0.97	0.87	0.33	0.94	1.00
**Practice loca- tion (urban)**	-0.04	-0.21	-0.13	**0.92**	-0.18	-0.02	0.41
	0.91	0.60	0.71	**0.04**	0.66	0.96	0.42
**Recall system CVD**^**5**^	-0.74	-0.93	-0.53	-0.76	-0.34	0.33	-0.60
	0.14	0.05	0.22	0.16	0.49	0.61	0.32
**E pt file**^**6**^	-0.48	-0.48	-0.75	**-2.14**	**-1.29**	-1.09	-0.92
	0.27	0.26	0.05	**0.00**	**0.00**	0.06	0.09
**Case finding methods**^**7**^	0.21	0.25	0.20	0.00	0.29	-1.19	0.84
	0.73	0.67	0.70	1.00	0.62	0.15	0.29
**Community resources**^**8**^	-0.06	-0.21	-0.07	-0.13	-0.07	-0.05	0.01
	0.70	0.15	0.58	0.42	0.63	0.82	0.96
**Education organisation**^**9**^	-0.03	0.10	0.22	**0.47**	0.12	0.00	0.09
	0.88	0.55	0.14	**0.01**	0.47	0.99	0.67
**List size**^**10 **^**linear**	3.99	3.16	2.99	**6.04**	2.44	**21.21**	1.19
	0.12	0.20	0.15	**0.04**	0.31	**0.00**	0.70
**List size quadratic**	-0.88	-0.72	-0.62	-1.19	-0.46	**-5.30**	-0.24
	0.13	0.19	0.19	0.07	0.40	**0.01**	0.72

Legend

^1^Marital status: 1= Married / cohabiting, 2= Single, Separated / divorced, Widowed.

^2^Self-assessment of health: How would you estimate your health status in general: 1= excellent, 2= very good and 3= good were aggregated to “good”, 4= fair and 5= poor to “poor”.

^3^Value of BMI (kg/m2).

^4^Yes/No answer of the patient if he/she has hypertension.

^5^Use of system for recall of people at risk for CVD.

^6^Computer-supported patient file system.

^7^If the practice uses case-finding methods to detect people with cardiovascular risk factors.

^8^Do practice physicians and nurses cooperate in local/community campaigns or actions?

^9^Education of providers: education of all nurses/GPs in the last five years on CVD, education of at least one nurse/GP on CVD in the last 15 months.

^10^Number of people on the patient list.

We could not find any statistical prediction of the following variables on received CVD advice: gender, employment, marital status, practice size, case-finding methods, community resources. Some variables were borderline statistically significant.

## Discussion

### Summary of main findings and their contextualisation

The lifestyle of the studied younger family practice population showed disappointing results regarding the level of physical activity, healthy food habits, the number of people who are currently regular or occasional smokers or who were smokers in the past and increased body weight/obesity. The figures on obesity were similar to the figures in a national epidemiological study, while the percentage of people exercising adequately showed a lower level of physical activity in our study [23].

In a group of healthy people 18–45 years of age (around 10% having isolated health problems like arterial hypertension or hypercholesterolemia), advice on any aspect of cardiovascular prevention was given in only 50% of the people.

Similar to other studies [24], we found that advice on physical exercise was the most common advice given (49.1%). Complex counselling on more aspects of lifestyle was also relatively common (45.3%). Percentages found in our study are slightly higher than in most of the other studies, where the figures in these studies were low in the early nineties [25], and higher in recent years [24] and varied considerably – advice on diet around 40% [26-28], on physical exercise 25-42% [26,27] and on smoking 30% or 40% for the adult population [27,29].

On the other hand, we found many »missing opportunities«, such as a low level of given written information or advice to visit educational websites. The latter might be especially important because this was a young generation, which is familiar with the internet and its information possibilities. Another less often provided advice was the advice on children’s lifestyle. In Slovenia, paediatricians are included in primary care and serve as children’s personal doctors. Regardless of this, family physicians should advise their patients – parents about children’s lifestyle too. Similarly, advice on community resources was not common, despite the fact that our country has a long tradition in community organisation of healthcare [30]. The percentage of advice on smoking was also surprisingly low, a classical field for education on healthy lifestyle. It was also perceived as the least helpful by respondents.

We found it very interesting to receive a stable result in the association of middle-sized patient lists with more regularly given advice for all types of healthy living lifestyles. Large list sizes of registered patients are an obstacle for preventive work, especially counselling [31]. Small list size practices are difficult to analyse, but they might experience difficulties in the organisation of preventive activities as the doctors from such practices might have been involved in other primary care services or do not work full-time in the practice.

Independent determinants for provided advice on managing blood cholesterol levels were the most numerous: patients’ weight and the following practice characteristics: urban location, practice education and patient list size. With only one exception (advice on cigarette smoking in respondents with poor self-assessment of health), we did not find any meaningful (or the significance was borderline) association with patient/practice characteristics and advice in three models: for cigarette smoking, weight and physical activity. More often, provision of advice on healthy lifestyle for respondents who assessed their health worse was also found in other studies, athough the results were not consistent across all types of advice [26]. On the contrary to our results, other studies found that a rural location was associated with better provision of preventive services [32].

Surprisingly, in the subsample of males from 35 to 45 years old, who are included in the Slovenian National Preventive Programme, our analyses could not confirm any higher figures on counselling for this group of people. This goes in line with other studies – the group of younger people with low risk of cardiovascular disease is known to be rather neglected in the provision of preventive activities [10,26].

We could not prove the positive relationship of electronic support system on advice; on the opposite, practices with computer-supported patient file systems gave less advice on several topics of lifestyle; we cannot explain the result but it can be connected with the lack of systematic recording in e-patient files.

The education of providers on preventive activities increased counselling on cholesterol. The result is interesting because it can show that education is too concentrated on secondary prevention and the management of risk diseases for CVD and does not emphasize lifestyle modification in general.

We can compare our results with the systematic review of Bock et al. [33]: some practice characteristics that were related to higher levels of counselling in the review were the size of the practice, protocols for prevention and printed materials in the practice. The first one was proven also in our study.

### Discussion on methodology

The methodology has already been tested in the pilot study for the EPA-Cardio study. The questionnaire for participants contained questions, which were the results of previous work in the EPA-Cardio study [34]. Validated questionnaires were used to obtain information on diet, physical activity and smoking status. Questions on practice characteristics were based on previous research on practice assessment [35].

A further strength of this study is a large sample, a good response rate and multi-level statistical analyses. The response rate was actually one of the highest compared to other countries in the Epa-Cardio study.

The inclusion of the practices followed strict stratification rules and every practice that refused to participate was replaced by a practice from the same stratification field. This in fact could be a source of selection bias.

As we can observe in other studies, the number of women in the sample was substantially higher, which can be attributed to the effect of self-choice: women are more frequent attenders of the practice than men [36] and more interested in participation in the survey than men [37]. Most of the respondents were long-term patients in the same practice, which is also in line with other European practices that have a patient list system [38].

We also have to address problems of multiple testing. The statistical models include many predictor variables: nine on the patient level and nine on the practice level. We do not, however, consider multiple testing to be an important issue as the inclusion of each predictor was theoretically well-grounded as described in the introduction.

The information on received advice came from the respondents and was not compared with their record and was therefore prone to subjectivity. We also have to be aware of possible social desirability in assessing the helpfulness of advice. However, other studies used the same methodology [39,40]. We also know that records on advice are not very consistent either [41], and that the agreement between self-reports and medical records varies [42,43]. The delivery and recording of advice was much higher in the systems with adequate incentives, such as in the Quality and Outcomes Framework programme in the United Kingdom [44,45]. In Slovenian paper or electronic records, counselling has no structured recording form and we believe that recording is forgotten on many occasions, although we do not have any statistical data to prove it. No incentives are given for the recording of counselling in this age group. Moreover, respondents’ information/perception of received advice is the information that we were looking for in this study.

As this was a cross-sectional study, we could not assess the effect of the advice given. We also did not include advice on alcohol consumption in the study.

### Recommendations for further research

As the research in the field of giving health advice to the young and healthy population is scarce, more studies are needed to determine the best possible methodology for providing advice in order to achieve maximum effectiveness. Also, there is a need for larger international studies in this subject, which will use prospective methodology to assess the success of such advice.

## Conclusion

In our study we could relate some people and practice characteristics to certain forms of cardiovascular prevention advice provided. It seems that basic counselling for lifestyle (smoking, weight and physical activity) is not provided widely enough to the younger population and we could prove only some associations in each model. It seems that the providers’ education is still too concentrated on what they perceive as a disease. The recording of advice in electronic files needs to be standardized and carried out systematically. Policy makers should be aware that list size matters in the uptake of preventive activities. Neither too small nor to large practices perform on a desirable level regarding provision of advice on healthy lifestyle for young people.

## Competing interests

All authors declare that they have no competing interest.

## Authors’ contributions

DP participated in the design of the study, acquisition and interpretation of the data and drafted the manuscript. RP performed the statistical analysis and interpretation and participated in manuscript writing. ZKK participated in the acquisition of the data and manuscript writing. JK coordinated the study, participated in the acquisition and interpretation of the data and manuscript writing. All authors read and approved the final manuscript.

## Pre-publication history

The pre-publication history for this paper can be accessed here:

http://www.biomedcentral.com/1471-2296/14/82/prepub
